# Investigating cognitive-enhancing supplement use among students at a Dutch life science university

**DOI:** 10.1371/journal.pone.0332433

**Published:** 2025-10-09

**Authors:** Elena Escano, Nilüfer Sezer, Annemieke Maria Pustjens

**Affiliations:** Wageningen Food Safety Research, Wageningen University and Research, Wageningen, The Netherlands; Health Researcher, SPAIN

## Abstract

The use of cognitive enhancers among college students has drawn significant attention due to their impact on academic performance and health. This study investigates the prevalence, types, and motivations for cognitive-enhancing supplements (CES) use among students at Wageningen University & Research, as well as their perception of safety and authenticity aspects of CES. Data were collected from December 2023 to January 2024 via an online survey. The analysis was performed using R Statistical Language. Among the 288 students who participated (mean age = 23.1 years), 51.7% were aware of CES, with higher awareness among non-EU students (52.1%) compared to Dutch (20.5%) and other EU students (24.6%) (p = 0.001). Older students (40.5%) were more likely to consider using CES than younger ones (21.1%) (p = 0.005). CES usage was 11.5%, which is lower than in other European studies. Safety was a major concern, with participants trusting primarily scientific research but feeling inadequately informed about CES safety. Differences in authenticity concerns between users and non-users were noted (p = 0.003). The findings highlight the need for improved information and regulatory oversight regarding CES.

## Introduction

Cognitive enhancers, commonly referred to as ‘smart drugs’ or ‘nootropics’, encompass a broad category of substances, including both prescription medications and over-the-counter supplements. Over-the-counter supplements, such as vitamins, minerals, herbs, amino acids, and enzymes among others, are readily available for purchase at local drug stores [1].

The use of cognitive enhancers among university students has garnered cnsiderable attention in scientific and academic circles [2,3].These substances are typically used with the aim of improving cognitive performance and academic achievement [4]. Their growing popularity has sparked debate concerning their safety, prevalence, underlying motivations, associated factors, and potential impacts on individuals and society [5].

A study involving 1,572 Dutch university students examined the prevalence and patterns of cognitive enhancer use in The Netherlands [3]. This research offered valuable insights within a specific educational and cultural context. The authors noted that cognitive enhancers may have health implications beyond academic performance, underscoring the need for further investigation into their broadereffects. Subsequent studies have expanded on these findings, providing a broader international perspective on the use of cognitive enhancers among university students [6].

A comprehensive systematic review examined the global landscape of cognitive enhancer usage, synthesizing data from multiple studies to identify common trends and regional differences. Building on prior research conducted in The Netherlands [3], the review offers insightful information about the frequency and usage trends of cognitive enhancers in a variety of geographic locations worldwide [6]. These findings contribute to a deeper understanding of how cognitive enhancers are used across diverse educational and cultural environments and underscore the need for targeted interventions and policy measures to mitigate potential health risks.

Recent contributions to the literature have further enriched this understanding [7,8]. For instance, Monnet et al [2] conducted a qualitative study exploring university students’ experiences with prescription drugs for academic purposes, offering valuable insights into the attitudes, behaviours, and motivations related to the use of cognitive enhancers. Similar research in countries such as the UK, France, Romania, and Poland [2] has offered insightful, context-specific insights into the prevalence, driving forces, and related variables affecting college students’ use of cognitive enhancers.

Given the increasing amount of studies on the subject and the variety of socio-cultural contexts in which it is used, it is essential to conduct a comprehensive investigation into university students’ usage of cognitive enhancers [9]. This study aims to examine the prevalence, types, and motivations behind the use of cognitive-enhancing supplements among Wageningen University & Research (WUR) students, thereby contributing to a better understanding of this phenomenon and its implications for academic institutions and public health policy. This integrated approach not only extends existing research but also highlights the importance of authenticity in addressing this issue.By exploring this topic in greater depth, the study seeks to identify patterns, differences, and emerging trends, thereby enhancing our understanding of this complex phenomenon and informing future research, policy and public healthinitiatives.

## Methodology

### Study procedures and sample

#### Study timeline and ethics approval.

This study was conducted from November 27^th^ 2023 to January 22^nd^ 2024. The survey was conducted in English and received approval from the WUR Research Ethics Committee (Approval number 2023−11), ensuring compliance with the Dutch Code of Ethics for research involving human participants in the social and behavioral sciences.

#### Participant invitation and informed consent.

Participants were invited to the study with a clear explanation of its goals. The survey aimed to gather information about the use of cognitive-enhancing supplements (CES), including motivations for consumption and commonly used types.

An online landing page provided detailed information, emphasizing voluntary participation, anonymity, and the right to withdraw without consequences. Written informed consent was obtained when participants initiated the survey. Participants were informed that participation would take between 2 and 10 minutes.

#### Recruitment and sample size.

Students at Wageningen University and Research (WUR) were recruited via flyers with QR codes distributed in student buildings and surroundings (i.e., student living complexes, and sports centers). The survey was conducted using the Qualtrics XM Platform (London, UK). The participants were also informed that they could win a voucher by providing their e-mail address, which was stored separately from the survey data. The final sample comprised 288 students.

### Definitions and measures

#### Cognitive-enhancing supplements.

To ensure a common understanding of CES among all participants, we provided a definition on the first page of the survey. This was done to guarantee that all respondents completed the questionnaire with a consistent comprehension of the topic. In this study, CES refers to ‘supplements taken with the intention of enhancing memory, mental alertness, concentration, energy levels, and wakefulness.’

#### Survey design and flow.

Participants were directed to a landing page where they were informed about the characteristics of the survey.

The survey was structured into distinct sections, with the initial questions focused on gathering demographic information such as sex, age, nationality (The Netherlands, other EU-countries, non-EU-countries), and level of education (BSc, MSc, or PhD). Subsequently, the survey assessed participants’ awareness, attitudes, and perceptions of CES use. The next section of the survey examined participants’ usage of CES, followed by questions pertaining to safety and legitimacy. In this final section, participants responded using a 7-point Likert scale, with 1 indicating strong agreement and 7 indicating strong disagreement. This structure allowed for a comprehensive understanding of participants’ knowledge, attitudes, and behaviors related to CES use (See S1 File).

The survey was developed based on inspiration from previous studies cited in the literature, with all authors actively participating in its validation through three iterative rounds of review. This process ensured the survey’s validity and relevance to our research objectives.

### Statistical analysis

The statistical analyses were performed in R Statistical Language (version 4.3.2; R Core Team, 2023). Descriptive statistics were calculated, including frequencies, means, and standard deviations. To assess the significance of associations between categorical variables, such as reported use of CES by gender or age, the chi-square and Fisher’s exact tests for count data were employed. The significance level is established at p < 0.05. For group comparisons, Chi-square tests were used where group population (n) was higher than 5; otherwise, Fisher’s Exact Test was applied.

All our findings are discussed in the context of broader trends and existing literature, rather than being treated as definitive based on their statistical significance.

## Results and discussion

### Participant description

A total of 288 students were included in the analysis for this study. The raw data are available in S2 File. Participants were selected based onhaving completed all demographic questions and indicating prior knowledge of cognitive enhancers. Although more participants initially began the survey, only those with complete data were retained in the final analysis. Participants ranged in age from 17 to 56 years, with an average of 23.1 years (SD = 3.5). For analytical purposes, respondents were divided into two age groups, those born before 2000 and those born in 2000 or later. The distribution of gender, level of current educational program (BSc, MSc, PhD), and country of origin (The Netherlands, other EU-countries, non-EU-countries) is presented in Table 1.

**Table 1:** Participant characteristics (n = 288).

	Participants (n)	Participants (%)
Gender Male Female Prefer not to say^*^	77 206 5	26.7 71.5 1.7
Current level of education BSc MSc PhD Other (pre-master)	120 143 23 2	41.7 49.7 8.0 0.7
Age Age ≥ 24 Age < 24	111 177	38.6 61.4
Origin Netherlands Other EU countries Non-EU	200 40 48	69.4 13.9 16.7

*Participants who selected “prefer not to say” for the gender question were excluded from the gender-related analyses.

### Awareness of cognitive enhancers

When assessing participants’ prior knowledge and awareness of CES, we found that 51.7% of the study population reported having heard of CES (Table 2). A statistically significant difference was observed based on the particpants’ origin (p = 0.001). Specifically, 20.5% of Dutch students and 24.6% of students from other EU countries were familiar with CES, compared to a notably higher percentage among students originating from non-EU countries (52.1%). No significant differences were found across gender, age groups, or education level.

**Table 2:** Awareness of cognitive-enhancing supplements (CES).

	n	Participants answering yes (%)	Participants answering no (%)	Participants answering not sure (%)^a^	Sign difference between sex	Sign difference between age^b^	Sign difference between education level	Sign difference between origin
Heard about CES	288	51.7	28.5	19.8	X-squared^c^ = 7.67 × 10 ⁻ ³, df = 2, p-value = 0.962	X-squared = 2.15 x 10^−2^, df = 2, p-value = 0.989	X-squared = 2.08, df = 4, p-value = 0.720	X-squared = 18.3, df = 4, p-value = 0.00108
Considered using CES yourself	204	28.9	71.1	n.a.	X-squared = 4.61 x10^-4^, df = 1, p-value = 0.983	X-squared = 7.89, df = 1, p-value = 0.005	X-squared = 3.95, df = 2, p-value = 0.138	X-squared = 12.7, df = 2, p-value = 0.001
Think it’s taboo to use CES	205	36.6	63.4	n.a.	X-squared = 0.80, df = 1, p-value = 0.370	X-squared = 0.50, df = 2, p-value = 0.780	X-squared = 11.3, df = 2, p-value = 0.003	X-squared = 6.34, df = 2, p-value = 0.041

^a^When given the option “not sure”; n.a.: not applicable.

^b^Two age groups were compared: participants born before 2000 (24 years and above) versus participants born after 2000 (below 24).

^c^Results from Chi square test. Df = degrees of freedom; p < 0.05 is considered significant.

Respondents who indicated that they had heard of CES or were unsure about their awareness, were presented with a multiple-response question to identify the sources through which they had obtained the information. The most frequently reported sources were family and friends (29.4%), the internet (26.6%), and social media (25.2%). These findings are consistent with a previous study conducted among university students in the United Arab Emirates [6], and align with results from studies in Switzerland and New Zealand, which similarly found that friends and social network were primary sources of information regarding CES [10]. Additional reported sources include pharmacies, medical books, commercials on TV, news, documentaries, TV shows, and podcasts.

When asked whether they had ever considered using CES themselves, 28.9% of the participants responded affirmatively. Among students aged 24 and older, 40.5% reported having considered using CES – significantly higher than the 21.1% reported by students younger than 24 (p = 0.005). To our knowledge, this age-related difference has not been reported before. It could be influenced by the fact that older students simply had more years of life to consider CES use.

A significant difference was also observed by country of origin: only 22.3% of Dutch students had considered using CES, compared to 48.4% of students from other EU countries and 47.8% from non-EU countries (p = 0.002). No significant differences were found based on gender (p = 0.983).

Overall, 36.6% of the participants consider it is taboo to use CES. This perception was significantly more prevalent among PhD students (72.2%) compared to BSc (30.4%) and MSc students (34.6%) (p = 0.003). The perception of CES as taboo is consistent with findings by Sharif et al.‘s (2021), who identified societal stigma and cultural expectations as key factors influencing CES use. Both studies suggest that the availability of CES and the influence of social interactions affect students’ use of these substances. Many students learn about CES through their social circles, family, and friends, as well as online sources like the internet and social media. This indicates that social relationships and access to information play a significant role in shaping students’ attitudes and behaviors regarding CES use, emphasizing the important role of peer networks and digital information in shaping perceptions.

In addition, a significantly larger proportion of Dutch students (41.5%) viewed CES use as taboo, compared to students from other EU countries (22.6%) and non-EU countries (21.7%) (p = 0.042). This suggests that cultural norms and societal attitudes in the Netherlands may contribute to this perception, which could, in turn, explain the lower rates of CES consideration among Dutch students.

### Use rates of cognitive enhancers

Of the participants, 33 students (11.5%) reported having ever used CES (Table 3). No statistically significant differences in CES use were found based on gender (p = 0.154), age group (p = 0.122), education level (p = 0.513), or country of origin (p = 0.195). Given the significant differences observed in whether participants had heard about CES and whether they considered using the pills themselves, the lack of variation in actual usage rates across these demogaphic categories was somewhat unexpected. It is important to acknowledge that comparisons across studies are complicated by inconsistent definitions of CES. Some research focuses solely on pharmacological cognitive enhancers, while others include recreational substances like coffee, nicotine, or energy drinks. This inconsistency in definitions presents challenges for making accurate cross-study comparisons.

**Table 3:** Usage of Cognitive-enhancing supplements (CES).

	n	Participants answering yes (%)	Participants answering no (%)	Participants answering not sure (%)^a^	Sign difference between sex	Sign difference between age^b^	Sign difference between education level	Sign difference between origin
Have you ever used CES?	287	11.5	83.3	5.2	X-squared^c^ = 2.03, df = 1, p-value = 0.154	X-squared = 2.39, df = 1, p-value = 0.122	X-squared = 1.34, df = 2, p-value = 0.513	X-squared = 3.27, df = 2, p-value = 0.195
Have you had any medical prescriptions?	42	28.6	71.4	na	X-squared = 0.63, df = 1, p-value = 0.429 (too small)	X-squared = 0.11667, df = 1, p-value = 0.733	X-squared = 2.7085e-31, df = 1, p-value = 1 (too small)	contingency_table X-squared = 1.5926e-31, df = 1, p-value = 1

^a^When given the option “not sure”; n.a.: not applicable.

^b^Two age groups were compared: participants born before 2000 (24 years and above) versus participants born after 2000 (below 24).

^c^Results from Chi square test. Df = degrees of freedom; P < 0.05 is considered significant.

In comparison to some other studies from other European countries, the use rate currently found is relatively low. For instance, a Portuguese study reported 31.1% usage of prescribed and nonprescribed drugs [11], a Greek study reported 21.5% usage [12], and a Swiss study reported 22% [13].

Almost half of the users (47.6%) have used CES only less than one month. The remained usedCES over longer periods, varying from 1–6 months (11.9%), 6–12 months (14.3%), 12–24 months (9.5%) to over two years (16.7%). Regarding access, 28.6% of CES users reported having a medical prescription. The majority of these prescriptions were for methylphenidate (9 out of 12), fllowed by dexamfetamin (3 out of 12). Notably, none of the PhD students in the sample reported having a medical prescription. Compared to a previous Dutch study, this percentage is very high, since Schelle et al [3] reported only 1.7% prescription use. Other European studies reported similarly low figures; 2.2% in France and Romania [14], 6.9% in Portugal [11], and 12% in Switzerland [13].

On the other hand, Maier et al [10] reported an increase in non-medical use of prescription stimulants (e.g., methylphenidate, dexamphetamine) in the Netherlands, from 8.2% in 2015 to 13.5% in 2017. If this upward trend has continued linearly, a 28.6% usage rate by 2024 may be plausible. This potential increase highlights the importance of ongoing monitoring and more nuanced investigation into patterns of CES use, including distinctions between medically supervised and non-medical usage.

Among the participants without a prescription, the most frequently consumed substance was caffeine (n = 16), followed by other substances specified by participants (n = 11). Other commonly mentioned substances included cobalamin (vitamin B12, n = 9), amphetamine salt mixtures (n = 6), methylphenidate (n = 6), and modafinil (n = 5). Additional reported substances were guarana (n = 4), pyridoxine (vitamin B6, n = 3), and vinpocetine (n = 1). Furthermore, participants mentioned consuming Ashwagandha, bacopa, vitamin D, L-theanine, L-tyrosine, fish oil, and supplements containing Rhodiola rosea, thiamine, riboflavin, pyridoxine, folic acid, and cyanocobalamin. Other CES cited included ritalin, dexamphetamine, citicoline, and ginkgo. These results illustrate the wide variety of compounds being used, with caffeine emerging as the most frequently mentioned substance. Also regarding participants who do not have a prescription, acces to CES is mainly through a physical shop in the Netherlands (26.2%), an online shop (23.8%), via a friend (21.4%), and in a pharmacy (19.0%). Other studies report higher shares (up to half of the participants) for sourcing via a friend [6,10]. This discrepancy may be attributed to differences in scope; while those studies focused primarily on prescription stimulants, the present study includes a broader range of supplements.

**Fig 1:**
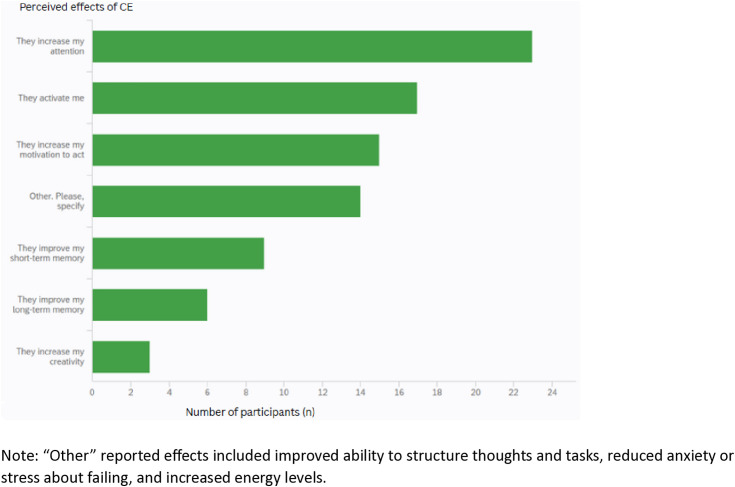
The answers to the question “What kind of pharmacological effects have you noticed during the use of cognitive-enhancing supplements (CES)?”.

Regarding the perceived effects of CES use, participants most frequently reported increased attention and a general sense of activation (Fig 1).Long-term memory and creativity are mentioned by the least number of students. Additional perceived benefits included enhanced ability to structure thoughts and tasks, reduced anxiety or stress about failing, and increased energy levels. Adverse effects that were mentioned are increased, e.g., heart rate, headaches, anxiety, sleeping problems, tremors, overstimulation, weight loss, increased blood pressure. Ram et al. [15] found that the most frequently cited negative outcomes of CES use were dependence and poorer physical health, with unwanted adverse effects ranking fourth in their study. Although dependence was not explicitly reported as a side effect in our study, the presence of symptoms such as anxiety and sleep issues may indicate a potential link to dependency on CES.

### Safety and legitimacy of CES use

**Fig 2:**
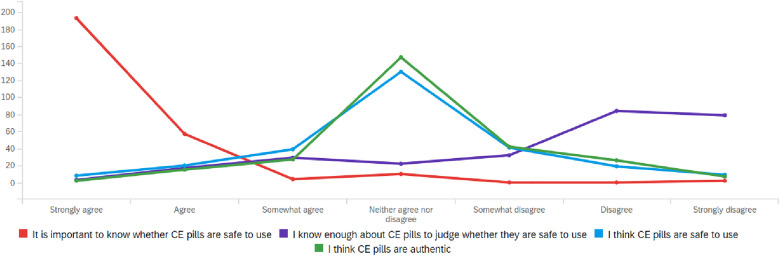
The answers to the question “To what extent participants agree to…?”. Responses were measured on a 5-point Likert scale ranging from ‘Strongly disagree’ to ‘Strongly agree’.

Beyond price, factors such as safety and authenticity of the CES could play a role in deciding whether or not to buy and use them. To explore this, participants were asked to score a number of these aspects on a 7-point scale. Results are presented in Table 4. Participants (n = 266) found it important to know whether CES are safe before use (average score of 1.4 and variance was low). However, when being asked whether they felt they had sufficient information to judge whether CES are safe, they gave an average score of 5.4 with a large variance. Apparently, there is quite some difference in this perception. When asked whether they think CES are safe to use, participants gave an average score of 4.0 (SD = 1.2), indicating a moderate level of uncertainty. As visualized in Fig 2, respondents consider it important to know whether CES are safe to use (red line), yet reported limited knowledge (purple line). Meanwhile, their trust in safety (blue line) and authenticity (green line) of CES was moderate. This gap between the perceived importance of safety and the lack of knowledge and trust may be cause for concern, as it reflects uncertainty about the quality and reliability of substances under consideration.No statistically significant difference in perceptions of safety were found based on gender, education level, and origin, and also not between CES users and non users. This is in contrast to the findings of Nguyen et al. [8], which suggest that users tend to believe that drugs are safer than they are perceived by non-users. However, both user and non-user groups in our study utilized comparable information sources and demonstrated similar, relatively limited drug safety knowledge.

**Table 4:** Safety and legitimacy of cognitive-enhancing supplements (CES) usage (n = 266).

	Mean score	Std deviation	Variance	95% CI	Sign difference between sex	Sign difference between age	Sign difference between education level	Sign difference between origin	Sign between consumption status
Importance to know CES are safe	1.40	0.85	0.72	1.30–1.50	p-value = 0.443	p-value = 0.548	p-value = 0.422	p-value = 0.327	p-value = 0.289
I know enough to judge whether CES are safe	5.37	1.62	2.64	5.18–5.56	p-value = 0.5718	p-value = 0.035	p-value = 0.559	p-value = 0.701	p-value = 0.289
I think CES are safe to use	4.01	1.21	1.46	3.86–4.16	p-value = 0.1248	p-value = 0.601	p-value = 0.108	p-value = 0.353	p-value = 0.125
I think CES are authentic	4.20	1.07	1.14	4.07–4.33	p-value = 0.4594	p-value = 0.963	p-value = 0.167	p-value = 0.271	p-value = 0.003

^a^Results from Fisher’s Exact Test for Count Data.

P < 0.05 is considered significant.

Note. The table reports mean scores, standard deviations (SD), variances, and 95% confidence intervals (CIs) for each item. p-values indicate results of significance tests across sex, age, education level, origin, and consumption status.

There are many different sources of information on the safety of CES. When considering the trust participants have, they trust scientific research the most (score 1.9 ± 0.9) and social media the least (score 5.4 ± 1.2). Personal experience (score 4.0 ± 1.6), experience of peers (score 3.9 ± 1.5), and information from websites (score 3.8 ± 1.4) scored in the intermediate range. Additional sources of information mentioned by participants included psychiatrist and other health care professionals, product suppliers, influencers, product labels, advertisements, and governmental organisations (such as the National Institute for Public Health and the Environment (RIVM)). One person answered that medicins in the EU are tested so much, that he/she has much trust in them. Another person checked specific websites from, e.g., the Netherlands pharmacovigilance centre Lareb (www.lareb.nl) and the socalled “pharmacotherapeutisch Kompas” by the National Health Care Institute (https://www.farmacotherapeutischkompas.nl).

Given the growing market for CES, these supplements have become increasingly vulnerable to fraudulent activities, such as the illicit addition of pharmaceuticals into herbal supplements to boost their effects and thereby sales [16]. Therefore, the participants were asked whether they think CES are authentic. The average score is 4.2 on the 7-point scale (SD = 1.1), indicating moderate trust. There was a significant difference between participants who use CES and those who do not (p = 0.003); users reported higher trust in the authenticity of these substances. They did not believe that their views on authenticity or safety were extreme in any direction, which is to be expected and supports the consistency of their responses. This aligns with existing literature on authenticity, where perceptions of safety and authenticity often remain moderate rather than polarized.

### Limitations of the study and recommendations for future research

Although the authors have tried their best in setting up a valuable study, there are also some limitations:

- The study relies on self-reported data, which may be subject to social desirability. Participants might underreport or overreport their use of CES due to stigma, legal concerns, or memory inaccuracies.
- The sample consists of students from a single university (Wageningen University & Research), which limits the generalizability of the findings to other student populations in the Netherlands or internationally. Cultural, institutional, and academic pressures may differ significantly across contexts. Future studies should include participants from multiple universities across different regions or countries.
- The recruitment strategy relied on voluntary participation through flyers posted in student areas. This method may have introduced self-selection bias, attracting individuals with a particular interest in or experience with cognitive enhancement. Additionally, the sample size may not have been sufficiently large to ensure adequate representation of the entire WUR student population. Thus, caution is warranted when generalizing the findings of this study.
- The cross-sectional nature of the study limits the ability to infer causality or understand changes over time. Longitudinal research would be necessary to assess how awareness, attitudes, or usage patterns evolve during students’ academic careers.
- The study includes a broad definition of cognitive enhancers, ranging from prescription stimulants to over-the-counter supplements and even caffeine. This may introduce inconsistencies when comparing results with studies that focus exclusively on pharmacological substances.
- While use duration is briefly reported, there is limited information about dosage, frequency, or context of use (e.g., during exams vs. regular use). This makes it difficult to assess the actual intensity and risk of CES use.
- Subgroups such as PhD students, users with medical prescriptions, or specific country-of-origin categories may have had small sample sizes, limiting the statistical power and reliability of subgroup comparisons.
  - Although participants rated aspects like safety and authenticity, their interpretations of these terms may vary. For example, some may equate “authentic” with legality, while others may interpret it as purity or manufacturer transparency. Qualitative studies or mixed-methods designs could explore how students assess the safety, authenticity, and ethical implications of CES use. Understanding decision-making processes may inform targeted interventions and health communication strategies.
  - The study does not account for confounding variables such as academic pressure, mental health status, or previous experiences with medication, which may influence CES use and attitudes. Further research should examine the role of academic pressure, stress, mental health conditions, and performance anxiety as potential drivers of CES use. Including validated psychological scales could yield deeper insights into these associations. Since our study found notable differences in perceptions of taboo and acceptability across cultural groups, future research should further explore how social norms, peer influence, and stigma shape students’ attitudes and behaviors regarding CES use.

Despite these limitations, the study provides valuable insights into the awareness, attitudes, and behaviors surrounding cognitive enhancer use in a university setting, and it highlights key areas for further research and policy attention. Next to the issues mentioned above, laboratory testing of CES products reported by users would provide critical insight into the authenticity and safety of these substances. This could help bridge the gap between perceived and actual risks, especially in unregulated markets.

Furthermore, our findings underscore the need for universities to enhance health education initiatives aimed at increasing awareness and promoting safer practices regarding CES use. By addressing the gaps in safety knowledge and reliance on unverified sources, universities can play a pivotal role in mitigating potential risks associated with the misuse of these substances.

## Conclusion

This study highlights the varying levels of awareness and attitudes towards cognitive-enhancing supplements (CES) among university students. While over half of the participants had heard of CES, actual usage was reported at 11.5%, which is lower than in other studies. Among Dutch students and those from other EU countries, 20.5% and 24.6% respectfully, were aware of CES, compared to a significantly higher rate of 52.1% among students from non-EU countries. Most students reported learning about CES through family and friends, the internet, and social media. Among participants without a prescription, the most common sourced for obtaining CES were physical shops, friends, and pharmacies. Safety concerns were prominent, while participants expressed trust in scientific research, many felt inadequately informed about CES safety. These findings underscore the need for improved education and regulatory oversight to address both safety issues and societal perceptions of CES use. Furthermore, they emphasize the importance of testing CES for authenticity to ensure that students are using safe and accurately identified substances—an essential consideration for protecting public health.

## Supporting information

S1 File Questionnaire. (PDF)

S2 File Individual results. (CSV)

## References

[1] Bochenek T, Godman B, Lipowska K, Mikrut K, Zuziak S, Pedzisz M, et al. Over-the-counter medicine and dietary supplement consumption among academic youth in Poland. Expert Rev Pharmacoecon Outcomes Res. 2016;16(2):199–205. doi: 10.1586/14737167.2016.1154790 26886826

[2] Monnet F, Ergler C, Pilot E, Sushama P, Green J. ‘Cognitive enhancers’: A qualitative exploration of university students’ experiences with prescription medicines for academic purposes. Policy Futures in Education. 2021;20(7):762–79. doi: 10.1177/14782103211061951

[3] Schelle KJ, Olthof BMJ, Reintjes W, Bundt C, Gusman-Vermeer J, van Mil ACCM. A survey of substance use for cognitive enhancement by university students in the Netherlands. Front Syst Neurosci. 2015;9:10. doi: 10.3389/fnsys.2015.00010 25741248 PMC4330699

[4] Napoletano F, Schifano F, Corkery JM, Guirguis A, Arillotta D, Zangani C, et al. The Psychonauts’ World of Cognitive Enhancers. Frontiers in Psychiatry. 2020;11. 33024436 10.3389/fpsyt.2020.546796 PMC7516264

[5] Ragan CI, Bard I, Singh I, Independent Scientific Committee on Drugs. What should we do about student use of cognitive enhancers? An analysis of current evidence. Neuropharmacology. 2013;64:588–95. doi: 10.1016/j.neuropharm.2012.06.016 22732441

[6] Sharif S, Guirguis A, Fergus S, Schifano F. The Use and Impact of Cognitive Enhancers among University Students: A Systematic Review. Brain Sci. 2021;11(3):355. doi: 10.3390/brainsci11030355 33802176 PMC8000838

[7] Lengvenyte A, Strumila R, Grikiniene J. Use of cognitive enhancers among medical students in Lithuania. Nordic Studies on Alcohol and Drugs. 2016;33(2):173–88. doi: 10.1515/nsad-2016-0014

[8] Nguyen NT, Rakow T, Gardner B, Dommett EJ. Understanding the relationship between safety beliefs and knowledge for cognitive enhancers in UK university students. PLoS One. 2021;16(1):e0244865. doi: 10.1371/journal.pone.0244865 33508011 PMC7842904

[9] Lanni C, Lenzken SC, Pascale A, Del Vecchio I, Racchi M, Pistoia F, et al. Cognition enhancers between treating and doping the mind. Pharmacol Res. 2008;57(3):196–213. doi: 10.1016/j.phrs.2008.02.004 18353672

[10] Maier LJ, Ferris JA, Winstock AR. Pharmacological cognitive enhancement among non-ADHD individuals-A cross-sectional study in 15 countries. Int J Drug Policy. 2018;58:104–12. doi: 10.1016/j.drugpo.2018.05.009 29902691

[11] Cavaco AM, Ribeiro J, Nørgaard LS. Exploring the use of cognitive enhancement substances among Portuguese university students. Explor Res Clin Soc Pharm. 2021;5:100097. doi: 10.1016/j.rcsop.2021.100097 35478516 PMC9032074

[12] Lazuras L, Ypsilanti A, Lamprou E, Kontogiorgis C. Pharmaceutical Cognitive Enhancement in Greek University Students: Differences Between Users and Non-Users in Social Cognitive Variables, Burnout, and Engagement. Subst Use Misuse. 2017;52(7):950–8. doi: 10.1080/10826084.2016.1267223 28426360

[13] Maier LJ, Liakoni E, Schildmann J, Schaub MP, Liechti ME. Swiss University Students’ Attitudes toward Pharmacological Cognitive Enhancement. PLoS One. 2015;10(12):e0144402. doi: 10.1371/journal.pone.0144402 26657300 PMC4675521

[14] Brumboiu I, Porrovecchio A, Peze T, Hurdiel R, Cazacu I, Mogosan C, et al. Neuroenhancement in French and Romanian university students, motivations and associated factors. International Journal of Environmental Research and Public Health. 2021;18(8):3880. 33917251 10.3390/ijerph18083880 PMC8068007

[15] Ram S, Hussainy S, Henning M, Stewart K, Jensen M, Russell B. Attitudes Toward Cognitive Enhancer Use Among New Zealand Tertiary Students. Substance Use & Misuse. 2017;52(11):1387–92. 28429997 10.1080/10826084.2017.1281313

[16] Paiva R, Correia M, Delerue-Matos C, Amaral JS. Adulteration of Brain Health (Cognitive, Mood, and Sleep Enhancement) Food Supplements by the Addition of Pharmaceutical Drugs: A Comprehensive Review of Analytical Approaches and Trends. Foods. 2024;13(6):908. doi: 10.3390/foods13060908 38540898 PMC10969376

