# Supplementary material for: Investigating cognitive-enhancing supplement use among students at a Dutch life science university
Source: PLoS One. 2025 Oct 9;20(10):e0332433. doi: 10.1371/journal.pone.0332433 (PMC12510594; doi:10.1371/journal.pone.0332433)
Supplement: S1 File — (PDF) [file pone.0332433.s001.pdf]

Survey Study Pills

Start of Block: Introduction

Introduction Dear participant,   Welcome to our survey about cognitive enhancer pills! Thank you for your interest in this survey designed by Wageningen Food Safety Research (WFSR), a research institute within WUR. There are several cognitive enhancement pills ("study pills") on the market, but it is not always clear what exactly they contain and whether they are safe to use. WFSR is developing an analytical tool that can identify potentially harmful or undisclosed compounds that may be present in cognitive enhancement pills. This project is part of the governmental program of WFSR, which is funded by the Ministry of Agriculture, Nature and Food Quality. The survey aims to gather information about the use of cognitive enhancer pills, including motivations for consumption and commonly used types. Your information will help us to better understand the usefulness and requirements of an analytical tool for these pills . The initial part of the survey involves filling in a consent form, after which you will proceed with the main part of the survey, where you'll be asked questions about study pills.   Your participation in this survey is entirely voluntary, and it should only take between 2 and 10 minutes to complete. Your responses will provide us with valuable insights.   As a gesture of gratitude for your participation, there is a chance to enter a lottery and win a €20 voucher upon completion of the survey. Thank you and good luck!

Page Break

Q0

This survey is being carried out by Wageningen Food Safety Research (WFSR) which is part of Wageningen University & Research (WUR). The purpose of this survey is to investigate the current use of cognitive enhancer pills ("study pills") among students. The results of this survey will help us to understand the usefulness and requirements for the development of a methodology for screening cognitive enhancement supplements to detect potentially harmful or undisclosed compounds. In this way, we hope to contribute to ensuring the safety and health of users of cognitive enhancer pills In addition, we give you the opportunity to participate in a lottery after finishing the survey. Five winners will be randomly selected to receive a voucher worth 20 euros each. To participate in the lottery following the survey, you will be asked for your email address (only to contact the winners), and your separate consent to participate in the lottery after finishing this survey. Information needed to give informed consent is provided at that point. The information collected from the survey and the lottery is processed and stored separately and never linked. You can participate in the survey without participating in the lottery. The survey requests the following information from participants: Demographic information (gender, age, geographical origin, and level of education), individual knowledge and perception about cognitive enhancer pills (awareness, personal reflection, attitude, and information origin), usage (usage history, prescription status, specific product used, timeframe, access, pharmacological effects, price influence, and compliance with recommended dosage) and safety & legitimacy (safety concerns, and trust in sources). WFSR will take utmost care to handle your data confidentially. Additionally, WFSR will not disclose any personal data to third parties. Only specific individuals involved in this project will have access to your data. By participating in this survey, you agree to the collection and storage of the outcomes of the survey for three years. Please note that individual answers provided in this survey will not be displayed or shared. All data is securely stored in accordance with the general guidelines of Wageningen University & Research Information security - WUR. To continue with the survey, we kindly request your confirmation that you have thoroughly read and understood the information provided above. By granting your voluntary and informed consent you agree to participate in the study. By giving your consent, you declare that you have provided this personal data voluntarily. The personal data you provide will only be used for the purpose for which you provided it. You have the right to inspect, delete, correct or limit the processing of personal data, as well as the right to object and the right to data portability. You have the right to decline to participate and withdraw from the research at any time, without any negative consequences, and without providing any reasons. More about this at Integrity and privacy - WUR. You will be given a unique session ID for this survey. We ask you to store that session ID for yourself (e.g. by writing it down or making a screenshot). If you want to exercise the above mentioned rights, you need to provide the session ID in your communication with us. Any questions about study participation can be directed to Nilüfer Sezer (nilufer.sezer@wur.nl). Any ethical concerns about the study can also be directed to Jacoline van der Zijden (rec@wur.nl) or Professor Moore, Chair of the WUR Research Ethics Committee at rec@wur.nl. Please note: you will be given a unique session ID for this survey. As we do not collect or store any directly identifying personal details, the session ID is the only way of recognizing you as a participant in

the survey. We ask you to store this session ID for yourself, and use it in your communication with us.

- ◯ Yes,I have read the above and agree to participate in the study (1)
- ◯ No (2)

Skip To: End of Survey If This survey is being carried out by Wageningen Food Safety Research (WFSR) which is part of Wagen... = No

Page Break

Q35 This is your ID number: ${e://Field/Random%20ID}

Q29 Are you currently enrolled as a student at WUR (Bachelor/Master/PhD)?

- Yes (1)
- No (2)

Skip To: End of Survey If Are you currently enrolled as a student at WUR (Bachelor/Master/PhD)? = No

Q1 What is your sex?

- Male (1)
- Female (2)
- Prefer not to say (3)

Q2 How old are you?

____

Q3 Where are you from?

- Netherlands (1)
- Other EU Countries (2)
- Non EU (3)

Q4 What is your current level of education?

- ○ BSc (1)
- ○ MSc (2)
- ○ PhD (3)
- ○ Other (4) ____

Page Break

End of Block: Introduction

Start of Block: Awareness

Q25 **What are cognitive enhancer pills(CE)?** Cognitive enhancer pills refer to pill supplements that individuals consume with the intention of enhancing memory, increasing their level of mental alertness and concentration, as well as elevating their energy levels and promoting wakefulness.

Q5 Have you ever heard about CE pills?

- No (1)
- Not sure (2)
- Yes (3)

Skip To: End of Block If Have you ever heard about CE pills? = No

---

Q6 Have you considered using any CE pill yourself?

- Yes (1)
- No (2)

Q7
Do you think it's taboo to use CE pills?

- Yes (1)
- No (2)

Q8 Where have you heard about CE pills?

- ☐ The Internet (1)
- ☐ Social media (2)
- ☐ Family and/or friends (3)
- ☐ Pharmacy (4)
- ☐ Medical books (5)
- ☐ Through commercials on tv (6)
- ☐ other. Please, specify (7) ____

End of Block: Awareness

Start of Block: Usage

Q9 Have you ever used CE pills?

- ○ Yes (1)
- ○ Not sure (2)
- ○ No (3)

Skip To: End of Block If Have you ever used CE pills? = No

Q30 Have you had any medical prescriptions for the use of these pills?

- ○ Yes. Please, specify (1) ____
- ○ No (2)

Q10 What CE pills have you used?

- ☐ Amphetamine salt mixtures (1)
- ☐ Methylphenidate (2)
- ☐ Modafinil (3)
- ☐ Piracetam (4)
- ☐ Caffeine (5)
- ☐ Cobalamin (vitamin B12) (6)
- ☐ Guarana (7)
- ☐ Pyridoxine (vitamin B6) (8)
- ☐ Vinpocetine (9)
- ☐ Other. Please specify (10) ____

Q12 How long have you taken the CE pills for?

- ○ Less than 1 month (1)
- ○ More than 1 month but less than 6 months (2)
- ○ more than 6 months but less than 1 year (3)
- ○ More than 1 year but less than 2 years (4)
- ○ 2 years or more (5)

Q15 How have you accessed CE pills?

- ○ Online-shop (1)
- ○ In a shop in The Netherlands (2)
- ○ In a shop abroad (3)
- ○ Via a friend (4)
- ○ Other. Please specify (5) ____

Q11 What kind of pharmacological effects have you noticed during the use of CE pills? You can mark more than one answer.

- ▢ They improve my short-term memory (1)
- ▢ They improve my long-term memory (2)
- ▢ They activate me (3)
- ▢ They increase my attention (4)
- ▢ They increase my motivation to act (5)
- ▢ They increase my creativity (6)
- ▢ Other. Please, specify (7) ____

Q16 What adverse effects have you noticed during the use of CE pills?

- ☐ Increased heart rate (1)
- ☐ Increased respiratory rate (2)
- ☐ Memory and concentration disturbances (3)
- ☐ Decreased tolerance to physical activity (4)
- ☐ Sleeping problems (5)
- ☐ Sausea (6)
- ☐ Vomiting (7)
- ☐ Diarrhea (8)
- ☐ Headaches (9)
- ☐ Overstimulation (10)
- ☐ Anxiety (11)
- ☐ Muscle aches (12)
- ☐ Tremors (13)
- ☐ Fatigue (14)
- ☐ other: Please specify (15) ____
- ☐ None (16)

Q17 Has price played a role in using a specific CE pill?

- ○ Yes (1)
- ○ No (2)

Q18 Have you closely followed the recommended dosage of this CE pill?

- ○ Yes (1)
- ○ No (2)

End of Block: Usage

Start of Block: Safety & legimaticy

Q21 Authenticity in CE pills refers to the product's genuineness and accuracy in terms of ingredients and dosages. It's crucial to ensure safety and effectiveness, as any deviation from the advertised ingredients or dosages could be harmful to the user.

Q22 How much do you agree with the following statements?

	Strongly agree (1)	Agree (2)	Somewhat agree (3)	Neither agree nor disagree (4)	Somewhat disagree (5)	Disagree (6)	Strongly disagree (7)
It is important to know whether CE pills are safe to use (1)	○	○	○	○	○	○	○
I know enough about CE pills to judge whether they are safe to use (2)	○	○	○	○	○	○	○
I think CE pills are safe to use (3)	○	○	○	○	○	○	○
I think CE pills are authentic (4)	○	○	○	○	○	○	○

Q24 How much do you agree with the following statement? I trust …… when considering the safety of CE pills

	Strongly agree (1)	Agree (2)	Somewhat agree (3)	Neither agree nor disagree (4)	Somewhat disagree (5)	Disagree (6)	Strongly disagree (7)
personal experience (1)	○	○	○	○	○	○	○
experience of peers (2)	○	○	○	○	○	○	○
Information from websites (3)	○	○	○	○	○	○	○
social media (4)	○	○	○	○	○	○	○
scientific research (5)	○	○	○	○	○	○	○
other (please specify) (6)	○	○	○	○	○	○	○

End of Block: Safety & legimaticy

Start of Block: Block 4

Q28 Thank you for completing our survey on the use of cognitive enhancer pills. We appreciate your participation and contribution to our research! **Please note that the survey you just completed has ended.**   However, if you would like to enter a lottery for a chance to win a prize, we invite you to click on the link below. We will ask you for your email address so that we can contact you in case you've received a reward. The lottery is preceded by an informed consent sheet, where we will explain how we will keep your e-mail address confidential. link   Please note that **the lottery is separated from the survey you just completed**, and your participation in the lottery is completely voluntary. Thank you again for your participation and contribution to our research!

End of Block: Block 4
